# Nomogram for predicting traumatic subdural effusion after mild traumatic brain injury

**DOI:** 10.3389/fneur.2022.947976

**Published:** 2022-09-01

**Authors:** Lichao Wei, Bowen Chang, Zhi Geng, Ming Chen, Yongsheng Cao, Liang Yao, Chao Ma

**Affiliations:** ^1^Department of Neurosurgery, The Third People's Hospital of Hefei, Anhui Medical University Hefei Third Clinical College, Hefei, China; ^2^Department of Neurology, The School of Mental Health and Psychological Sciences, The First Affiliated Hospital of Anhui Medical University, Anhui Medical University, Hefei, China; ^3^Division of Life Sciences and Medicine, Department of Neurosurgery, The First Affiliated Hospital of USTC, University of Science and Technology of China Hefei, Hefei, China; ^4^Department of Neurosurgery, School of Medicine, XinHua Hospital, Shanghai Jiaotong University, Shanghai, China

**Keywords:** nomogram, prediction model, risk factors, traumatic subdural effusion, mild traumatic brain injury

## Abstract

**Objective:**

Traumatic subdural effusion (TSE) is a common complication of traumatic brain injury (TBI). This study aimed to determine the risk factors associated with subdural effusion and to propose a nomogram to predict the risk of TSE in patients with mild TBI.

**Methods:**

We retrospectively analyzed 120 patients with mild TBI between January 2015 and December 2020 at the Third People's Hospital of Hefei. The risk factors of TSE were selected using univariate and multivariable logistic regression analysis. A nomogram was developed to predict the incidence of TSE. Receiver operating characteristics and calibration plots were used to evaluate the discrimination and fitting performance.

**Results:**

Of the 120 patients, 32 developed subdural effusion after mild TBI. Univariate analysis showed that gender, age, history of hypertension, traumatic subarachnoid hemorrhage, subdural hematoma, basilar skull fracture, and cerebral contusion were varied significantly between groups (*p* < 0.05). Logistic multivariate regression analysis showed that the gender, age, history of hypertension, and basilar skull fracture were independent risk factors for TSE. Based on these results, a nomogram model was developed. The C-index of the nomogram was 0.78 (95% CI: 0.70–0.87). The nomogram had an area under the receiver operating characteristic curve of 0.78 (95% CI: 0.70–0.87). The calibration plot demonstrated the goodness of fit between the nomogram predictions and actual observations.

**Conclusion:**

Gender, age, history of hypertension, and basilar skull fracture can be used in a nomogram to predict subdural effusion after mild TBI.

## Introduction

Traumatic brain injury (TBI) is a significant public health concern. It has high morbidity worldwide and is one of the most common diseases in neurosurgery (1, 2). Mild TBI accounts for more cases with craniocerebral injury than those with moderate and severe TBI, and it is often easily overlooked due to mild symptoms (3). Although the vast majority of patients with mild TBI recover completely within weeks or months without special treatment, 10–20% of patients still have post-traumatic syndrome (4).

Traumatic subdural effusion (TSE) is a common complication after TBI. The incidence of TSE is ~5–21% (5, 6). There are different reports on whether TSE affects the prognosis of patients, but there is no definite conclusion. Whether the formation of TSE has a greater impact on the prognosis of patients has yielded different conclusions in numerous studies. In a study by Kim et al. (7), it was believed that the occurrence of TSE was unrelated to a patient's prognosis, as it only prolongs the patient's hospital stay. However, in another study (5), TSE was divided into four types according to different clinical manifestations. The development type mainly manifested as progressive increased intracranial pressure, mild hemiplegia, aphasia, and abnormal mentality, meanwhile the patients with the development type often require surgical treatment and may die due to accompanying cerebral parenchymal damage or postoperative complications. Regardless of whether the occurrence of TSE affects the prognosis of the patient, some of the current confirmed theories are related to the occurrence of TSE. For example, subdural effusion may transform into a chronic subdural hematoma. Various countries and regions report different transformation rates of subdural effusion into chronic subdural hematoma after craniocerebral trauma, with a reported overall rate of 8–58%. Its transformation mechanism may be related to the repeated bleeding of new capillaries under the dura (8–10).

Several studies investigated the risk factors for TSE; however, these studies focused only on severe TBI in patients who underwent craniectomy (7, 11, 12). Therefore, it remains unknown whether patients with mild TBI have identical findings; and which clinical characteristics of patients with mild TBI may lead to TSE. This retrospective study analyzed the clinical data of 120 patients with mild TBI at Hefei Third People's Hospital. In addition, risk factors for TSE were explored. Finally, a prediction model was constructed to predict the incidence of TSE.

## Materials and methods

### Study population

Data were collected from 120 patients with mild TBI who were admitted to the Department of Neurosurgery of the Third People's Hospital of Hefei from January 2015 to December 2020. The inclusion criteria were as follows: (1) The patient admitted to hospitial with craniocerebral trauma and Glasgow Coma Scale (GCS) scores≥ 13. (2) The patient underwent cranial CT or MRI for the first time after admission, with no imaging findings of TSE; (3) The patient's head CT findings were reviewed before discharge or within 10 days of admission. The exclusion criteria were as follows: (1) the patient was admitted to the hospital because CT or MRI indicated TSE; (2) the patient died after admission; (3) the patient was not suitable for participation in the study due to other reasons. The diagnostic criteria of TSE: (1) The effusion appeared within 10 days of head trauma; (2) similar uniform low-density area and width >3 mm; (3) CT value of lesion area <20 Hu; (4) CT did not show any enhanced capsule.

This study was conducted in accordance with the Declaration of Helsinki and was approved by the Third People's Hospital of Hefei. As this study featured a retrospective design, the ethics committee judged it as minimal risk research and the participants could not be located. All study data of the participants were kept confidential by the investigators according to the guidelines of ethics.

### Data acquisition

General information, basic diseases and history of TBI were checked and recorded in all patients. The general information included the patient's gender, age, and basic diseases, including hypertension and diabetes. History of TBI data included GCS scores, traumatic subdural hematoma, traumatic subarachnoid hemorrhage, epidural hematoma, cerebral contusion, basilar skull fracture and whether mannitol was used.

### Statistical methods

Continuous variables are presented as mean ± standard deviation. Categorical variables are presented as numbers or percentages. The normal distribution of each variable was evaluated by the Kolmogorov-Smirnov test. For normally distributed data, we examined intergroup differences using the two-tailed Student's *t*-test. For non-parametric data, we examined intergroup differences using the Mann-Whitney *U-*test. We incorporated the related variables from the univariate analysis (*p* < 0.05) into the multiple regression analysis to avoid omitting important variables. Logistic regression multivariate analysis of variance was used to explore related risk factors. Subsequently, these risk factors were used to develop the regression model. The model was then transformed into a nomogram. Calibration plots were used to assess the model. Statistical significance was set at *P* < 0.05. Empower (R) (www.empowerstats.com, X&Y Solutions, Inc., Boston, MA, USA) and R 3.6.3 (http://www.R-project.org) were used for statistical analyses.

## Results

### Clinical characteristics

A total of 120 patients diagnosed with mild TBI were enrolled in our study from January 2015 to December 2020. The demographic information and related risk factors are listed in Table 1. According to our study design, we divided our study cohort into the TSE group and the non-TSE group. The non-TSE group, included 34 females (38.6%) and 54 males (61.4%), with a mean age of 49.1 years. The TSE group, included 6 female (18.8%) and 26 male (81.2%), with a mean age of 60.9 years. Patient gender (*p* = 0.041), age (*p* = 0.001), history of hypertension (*p* < 0.001), GCS scores (*p* = 0.043), cerebral contusion (*p* = 0.016), subarachnoid hemorrhage (*p* = 0.015), acute subdural hematoma (*p* = 0.028), and basilar skull fracture (*p* = 0.016) were significantly different between the TSE and non-TSE group. Table 2 shows the results of the univariate analysis of the various variables. The results show that TSE was associated with age (*p* = 0.0025), gender (*p* = 0.046), history of hypertension (*p* = 0.0007), cerebral contusion (*p* = 0.0408), subarachnoid hemorrhage (*p* = 0.0174), acute subdural hematoma (*p* = 0.0376), and basilar skull fracture (*p* = 0.0312). Table 3 shows the results of the multivariate regression analysis of various variables between TSE and non-TSE groups. Factors that were statistically different in univariate analysis included age, gender, history of hypertension, cerebral contusion, subarachnoid hemorrhage, acute subdural hematoma and basilar skull fracture. The presence or absence of TSE was used as a binary-classification-dependent variable. Furthermore, the multivariate regression analysis showed that the gender (OR = 0.23, 95% CI: 0.06–0.82, *p*=0.0239), age (OR = 1.03, 95% CI: 1.00–1.06, *p* = 0.0458), history of hypertension (OR = 3.64, 95%CI: 1.15–11.49, *p* = 0.0278), basilar skull fracture (OR = 5.25, 95% CI: 1.66–16.67, *p* = 0.0049) were independent risk factors for the evolution of TSE.

**Table 1:** The baseline characteristics of mild traumatic brain injury patients.

**Variables**	**No**	**Yes**	**Standardize diff**.	***P*-value**
**N**	88	32		
**Age**	49.14 ± 17.48	60.88 ± 16.82	0.68 (0.27, 1.10)	0.001
**GCS scores**	14.93 ± 0.33	14.75 ± 0.62	0.36 (-0.04, 0.77)	0.042
**Gender**			0.45 (0.04, 0.86)	0.041
Male	54 (61.36%)	26 (81.25%)		
Female	34 (38.64%)	6 (18.75%)		
**History of hypertension**			0.71 (0.29, 1.12)	<0.001
Yes	10 (11.36%)	13 (40.62%)		
No	78 (88.64%)	19 (59.38%)		
**ASDH**			0.41 (-0.00, 0.82)	0.028
Yes	5 (5.68%)	6 (18.75%)		
No	83 (94.32%)	26 (81.25%)		
**SAH**			0.54 (0.13, 0.95)	0.015
Yes	47 (53.41%)	25 (78.12%)		
No	41 (46.59%)	7 (21.88%)		
**EDH**			0.02 (-0.38, 0.43)	0.912
Yes	6 (6.82%)	2 (6.25%)		
No	82 (93.18%)	30 (93.75%)		
**Diabetes**			0.05 (-0.35, 0.46)	0.791
Yes	2 (2.27%)	1 (3.12%)		
No	86 (97.73%)	31 (96.88%)		
**Basilar skull fracture**			0.44 (0.03, 0.84)	0.028
Yes	14 (15.91%)	11 (34.38%)		
No	74 (84.09%)	21 (65.62%)		
**Mannitol used**			0.32 (-0.08, 0.73)	0.123
Yes	41 (46.59%)	20 (62.50%)		
No	47 (53.41%)	12 (37.50%)		
**Cerebral contusion**			0.58 (0.17, 1.00)	0.016
Yes	69 (78.41%)	31 (96.88%)		
No	19 (21.59%)	1 (3.12%)		

GCS scores, Glasgow Coma Scale scores; ASDH, acute subdural hematoma; SAH, subarachnoid hemorrhage; EDH, epidural hematoma.

**Table 2:** Univariate analysis of patient characteristics and TSE occurrence.

**Variables**	**Statistics**	**TSE OR (95% CI)**	***P*-value**
**Gender**			
Female	40 (33.33%)	0.37 (0.14, 0.98)	0.0460
Male	80 (66.67%)	1.0	
**Age**	52.27 ± 18.01	1.04 (1.01, 1.07)	0.0025
**GCS scores**	14.88 ± 0.43	0.44 (0.18, 1.03)	0.0597
**History of hypertension**			
Yes	23 (19.17%)	5.34 (2.03, 14.01)	0.0007
No	97 (80.83%)	1.0	
**ASDH**			
Yes	11 (9.17%)	3.83 (1.08, 13.59)	0.0376
No	109 (90.83%)	1.0	
**SAH**			
Yes	72 (60.00%)	3.12(1.22, 7.95)	0.0174
No	48 (40.00%)	1.0	
**EDH**			
Yes	8 (6.67%)	0.91(0.17, 4.76)	0.9122
No	112 (93.33%)	1.0	
**Diabetes**			
Yes	3 (2.50%)	1.39(0.12, 15.84)	0.7923
No	117 (97.50%)	1.0	
**Basilar skull fracture**			
Yes	25 (20.83%)	2.77 (1.10, 6.99)	0.0312
No	95 (79.17%)	1.0	
**Mannitol used**			
Yes	61 (50.83%)	1.91 (0.83, 4.38)	0.1260
No	59 (49.17%)	1.0	
**Cerebral contusion**			
Yes	100 (83.33%)	8.54 (1.09, 66.64)	0.0408
No	20 (16.67%)	1.0	

GCS scores, Glasgow Coma Scale scores; ASDH, acute subdural hematoma; SAH, subarachnoid hemorrhage; EDH, epidural hematoma.

**Table 3:** Multivariate logistic regression analysis of risk factors for TSE.

**Variables**	**TSE OR (95% CI)**	***P*-value**
**Age**	1.03 (1.00, 1.06)	0.0451
**Gender**		
Female	0.24 (0.07, 0.88)	0.0310
Male	1.0	
**History of hypertension**		
YES	3.78 (1.18, 12.10)	0.0249
No	1.0	
**ASDH**		
Yes	2.79 (0.55, 14.22)	0.2169
No	1.0	
**SAH**		
Yes	1.0	0.1324
No	2.71 (0.74, 9.91)	
**Basilar skull fracture**		
Yes	4.97 (1.56, 15.90)	0.0068
No	1.0	
**Cerebral contusion**		
Yes	0.90 (0.08, 10.55)	0.9344
No	1.0	

ASDH, acute subdural hematoma; SAH, subarachnoid hemorrhage.

### Development of the nomogram

**Figure 1:**
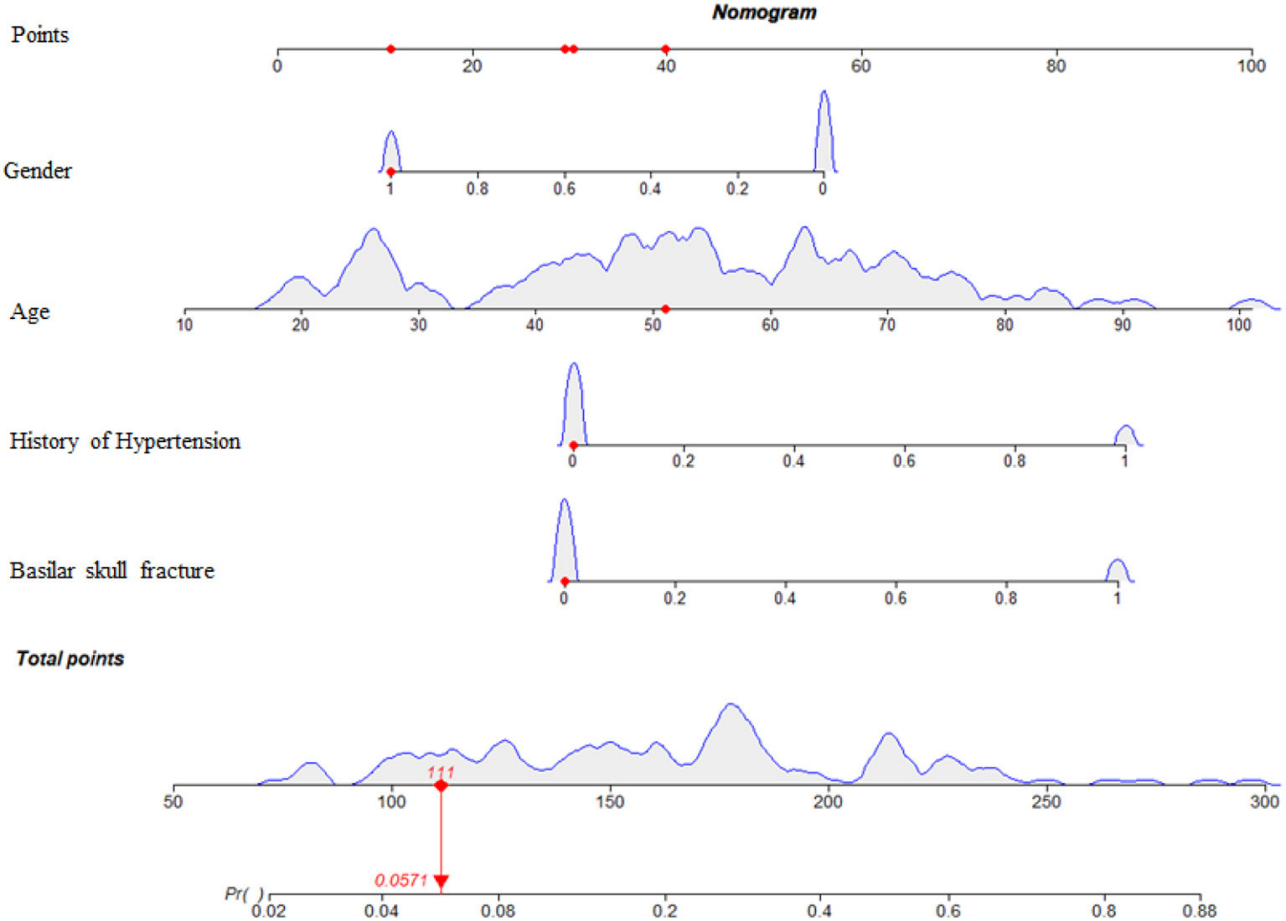
Nomogram predicting the incidence probability of TSE. For Gender, 0 = male, 1 = female. For history of hypertension, 0 = no, 1 = yes. For Basilar skull fracture, 0 = no, 1 = yes.

**Figure 2:**
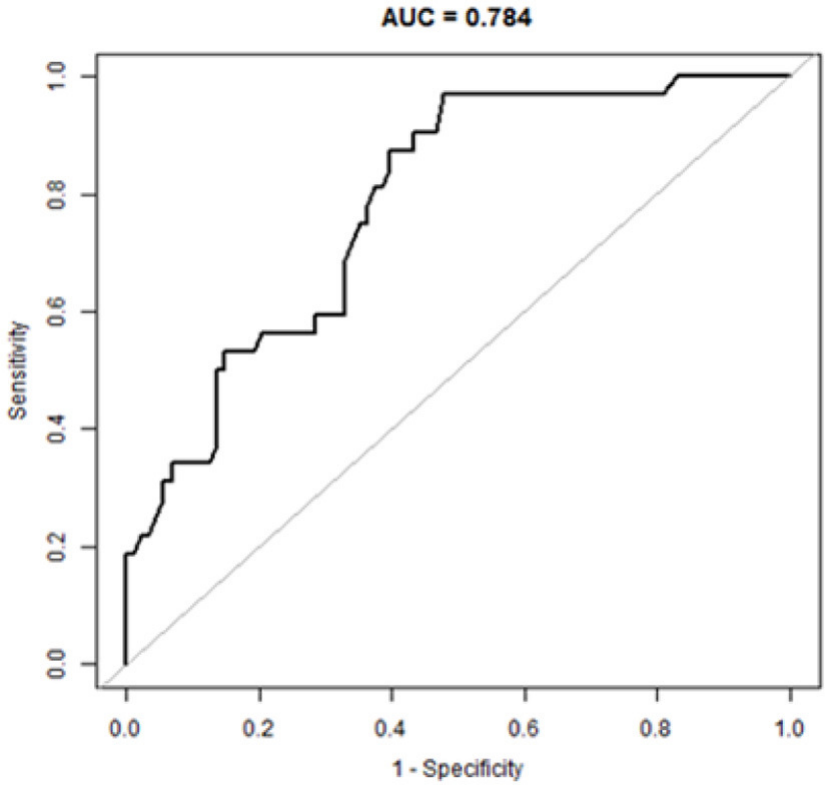
A receiver operating characteristic curve to evaluate the discriminating capability of the nomogram.

Based on these results, we developed a predictive model and generated a nomogram to predict the incidence of TSE (Figure 1). Each clinical factor corresponds to a specific score, and a straight line is drawn up to the point axis to calculate the total score, which corresponds to the probability of TSE occurrence. Figure 2 demonstrates that the prediction model had a fairly good discriminatory ability, with an area under the ROC curve of 0.78 (95% CI: 0.70–0.87). The C-index for TSE prediction model was 0.78 (95% CI: 0.70–0.87).

### Validation of the nomogram

**Figure 3:**
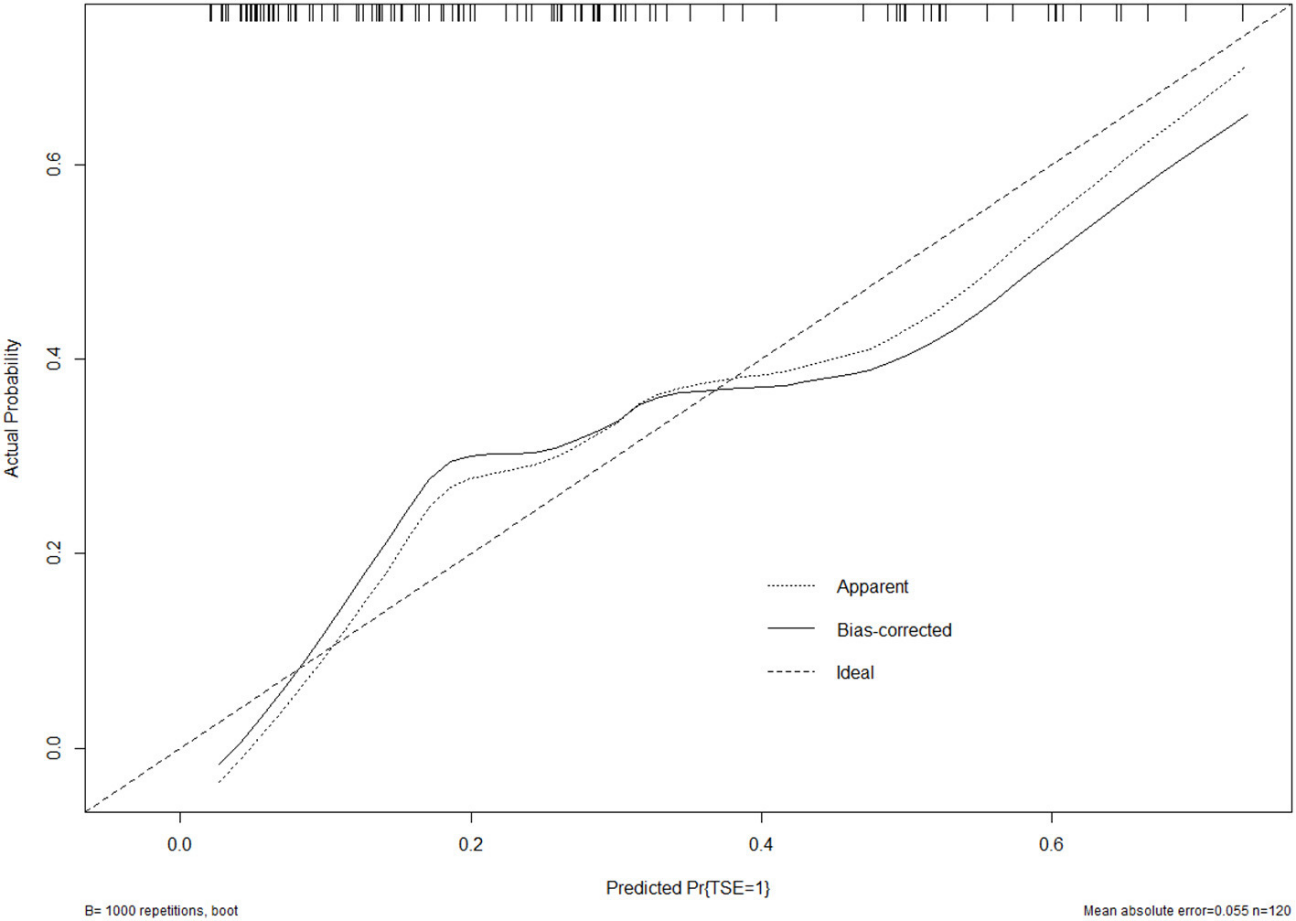
Calibration curve of the model. The calibration of the model in line with the agreement between predicted and observed outcomes of TSE.

The bootstrap validation method was used for internal validation of the generated model with a bootstrap-corrected C index of 0.75. Moreover, a calibration curve was generated by plotting the actual diagnosed TSE (Y-axis) against the predicted incidence risk (X-axis). The results in Figure 3 show a good degree of fit between the predictions and observations.

## Discussion

This was the first study to develop a nomogram to predict the incidence of TSE through investigating risk factors for TSE in a large cohort of patients with mild TBI. In this study, 120 patients with TBI were included to analyze the relevant factors that may affect the occurrence of TSE. The study found that the formation of TSE may be related to a patient's history of hypertension, traumatic subarachnoid hemorrhage, subdural hematoma, cerebral contusion, or basilar skull fracture. In this study, these factors were investigated further. Through multivariate logistic regression analysis, we found that the patient's gender, age, history of hypertension, and basilar skull fracture were risk factors for the formation of TSE.

Accumulation of cerebrospinal fluid (CSF) in the subdural space after TBI is called TSE which is a common complication of craniocerebral trauma (13). At present, there are different theories of the pathogenesis of TSH; however there is still controversy. The one-way valve theory states that: after TBI, intracranial brain tissue is displaced in the cranial cavity, causing the arachnoid to be torn, forming a one-way valve opening, and leading to the CSF then flowing into the subdural space along the valve opening. The blood-brain barrier destruction theory states that: after craniocerebral injury, the blood-brain barrier is destroyed, the permeability of capillaries is enhanced, and a large number of plasma leaks into the subdural space to form subdural effusion. The theory of intracranial pressure imbalance states that: after a patient undergoes TBI or post-traumatic surgical treatment, the pressure in the cranial cavity is unbalanced, causing CSF to accumulate on the side with lower pressure, changing the dynamics of CSF and forming subdural effusion (14, 15).

Many studies have indicated that age is not a risk factor for patients with severe TBI who have undergone decompressive craniectomy (6, 7, 11). However, studies have shown that older patients are prone to subdural effusion for without decompressive craniectomy (16). Haines et al. (17) found that the dura mater and arachnoid are composed of a limbic cell layer and an arachnoid barrier cell layer. The limbic cell layer is in the inner layer of the dura mater and is composed of fibroblasts; however, it contains very few collagen fibers, and the cells are less connected; and the arachnoid barrier layer is located in the outer layer of the arachnoid. This structure is fragile and can easily be torn in a TBI, creating a space between the dura mater and arachnoid. It is well-known that the condition for the formation of subdural effusion is that there is a potential space between the dura mater and the arachnoid. Because older patients often have brain atrophy, the subdural space is relatively large; after head trauma, this space is more conducive to the formation of TSE between the dura mater and arachnoid space. This may explain why age was one of the risk factors for the formation of TSE in our study. Therefore, for older patients, even if the clinical manifestations are not typical, CT or MRI findings should be reviewed promtly after injury for early detection and treatment of this complication.

Hypertension affects nearly one-third of the world's population and has become a global health threat (18). Studies have shown that hypertension can cause damage to the blood-brain barrier and increase cerebral vascular permeability (19). Additionally, TBI can disrupt the blood-brain barrier (20). Therefore, the damage to the blood-brain barrier is more likely to occur, and just as in the blood-brain barrier destruction theory, subdural effusion is more likely to form. In clinical work, when a patient has a history of hypertension, clinicians should be aware of the possibility of TSE.

In this study, males were more prone to TSE than were females, which may be related to the higher probability of trauma in males. In a study by Qiang Yuan et al. (16) it was found that males were more likely to have TSE than female. Although the incidence of TSE is higher, the reasons for this have not yet been reported in the literature. It may be related to the higher incidence of trauma in male.

Basilar skull fractures typically involve the areas of the temporal bone, occipital bone, sphenoid bone or ethmoid bone, which fractures can tear the arachnoid membrane or endocranium, resulting in leakage of CSF (21). When the dura mater and the arachnoid are ruptured at the same time, the CSF flows out along the fracture seam to form a CSF leak (22). It is possible that when the arachnoid ruptures and the dura mater is not ruptured, which forms a one-way valve opening, the arachnoid CSF flows out into the space between the dura and the arachnoid to form subdural fluid. Therefore, when patients with craniocerebral trauma are accompanied by skull base fractures, clinicians should be wary of the occurrence of TSE and actively prevent the progress of effusion. Early detection and early treatment may help prevent poor prognosis due to TSE.

Early detection is a key to preventing progression to TSE. This is best accomplished by identifying high-risk patients. Consequently, these patients need to be under observation, followed up, and should undergo dynamic-CT review. Early intervention and treatment are needed after TSE is found. The nomogram is one of the methods to present the predicted model as the graphic scoring. After performing the multivariate regression analysis, we included gender, age, history of hypertension and basilar skull fracture, a total of four factors, as nomogram score points. The model had good prediction ability, with a C index is 0.78 (95% CI: 0.70–0.87) and an area under the receiver operating characteristic curve of 0.78 (95% CI: 0.70–0.87). The calibration plot almost perfectly fits the actual situation. In clinical work, clinicians can directly see the possibility of TSE in patients with mild TBI through the nomogram.

This study had some limitations. First, this was a retrospective study and may therefore have a certain degree of selection and analytical bias. Second, only a limited number of patients were recruited from a single institution. It is necessary to conduct large sample and multicenter studies to prove the feasibility of the nomogram and increase the possibility of extensive popularization of the model.

## Conclusion

In summary, the current study found that gender, age, history of hypertension, and basilar skull fractures were independent risk factors for TSE. Based on these results, a nomogram model was developed. In addition, the ROC curve and calibration plot were used to show that the nomogram had a good predictive performance and calibration. Therefore, it may be of great value to facilitate the individualized prediction of TSE to reduce the risk of harm to patients.

## Data availability statement

The raw data supporting the conclusions of this article will be made available by the authors, without undue reservation.

## Author contributions

LW designed and conceptualized the study and drafted the manuscript. YC, CM, and LY helped to collect the data. ZG helped to collect the data and supervised the study. BC and MC designed the study, supervised the study, and revised the manuscript for intellectual content. All authors contributed to the article and approved the submitted version.

## Conflict of interest

The authors declare that the research was conducted in the absence of any commercial or financial relationships that could be construed as a potential conflict of interest.

## Publisher's note

All claims expressed in this article are solely those of the authors and do not necessarily represent those of their affiliated organizations, or those of the publisher, the editors and the reviewers. Any product that may be evaluated in this article, or claim that may be made by its manufacturer, is not guaranteed or endorsed by the publisher.
